# Rare case of early squamous cell carcinoma extending from the cardia to the fundus cured by endoscopic submucosal dissection

**DOI:** 10.1055/a-2277-0462

**Published:** 2024-03-20

**Authors:** Tingzhu Lan, Hui Xie, Xianzong Ma, Lang Yang, Peng Jin

**Affiliations:** 1 617516 Gastroenterology, The Seventh Medical Center of PLA General Hospital, Beijing, China; 2 Gastroenterology, Longgang District Central Hospital of Shenzhen, Shenzhen, China; 3 104607 Medical School of Chinese PLA, Beijing, China; 4 Senior Department of Gastroenterology, The First Medical Center of Chinese PLA General Hospital, Beijing, China

**Fig. 1:**
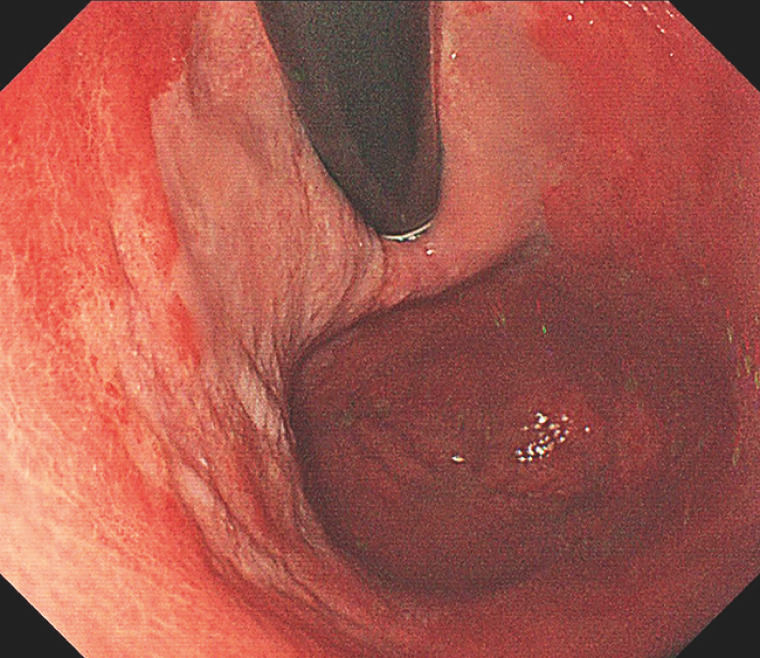
Squamous metaplasia extending from the cardia to the fundus of the stomach (white area).

**Fig. 2:**
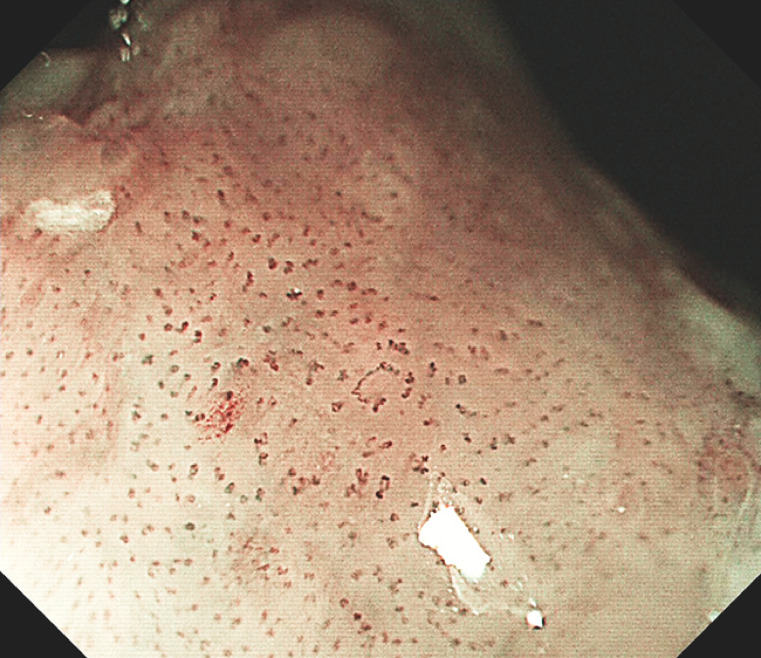
Narrow-band imaging revealed a brown area and abnormal intraepithelial papillary capillary loops within the squamous metaplasia of the gastric mucosa.

**Fig. 3:**
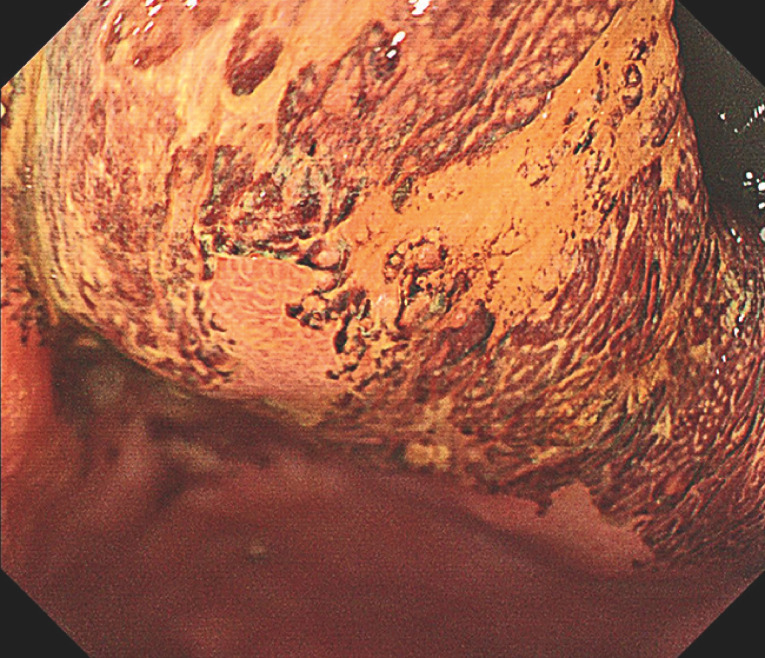
Lugol’s iodine staining showed a clear unstained lesion within the dark brown mucosa.

**Fig. 4:**
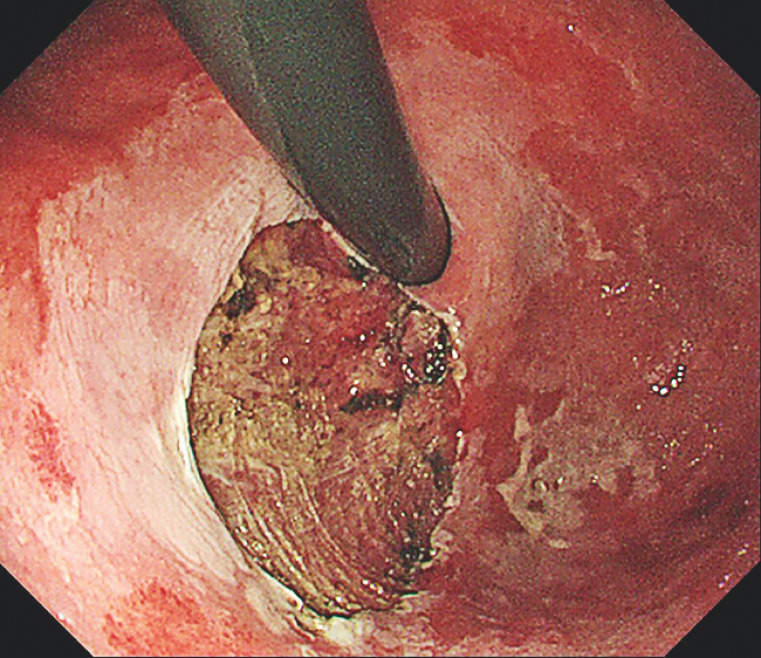
Appearance after complete lesion resection by endoscopic submucosal dissection.

**Fig. 5:**
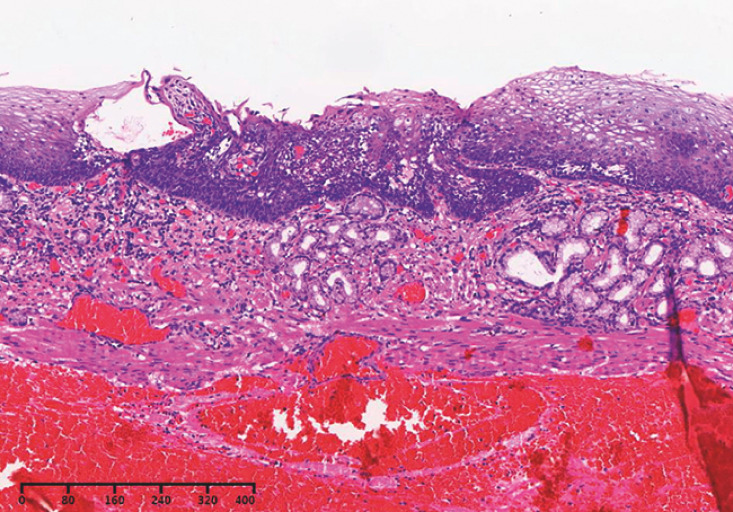
Histopathology confirmed an intramucosal squamous cell carcinoma infiltrating into the lamina propria.

A 70-year-old asymptomatic man with a preference for hot food and chronic alcohol consumption presented to our hospital for screening esophagogastroduodenoscopy, which revealed squamous metaplasia extending from the cardia to the fundus of the stomach ( Fig. 1 ). Narrow-band imaging and magnified endoscopy showed a brown area and abnormal intraepithelial papillary capillary loops of type B1 within the squamous metaplasia of the stomach mucosa ( Fig. 2 ), suggestive of early squamous cell carcinoma (SCC) with a low risk of submucosal infiltration. Biopsy confirmed high-grade dysplasia of the squamous epithelium. Endoscopic submucosal dissection (ESD) was performed to resect the lesion ( Video 1 ). Lugol’s iodine staining was used to determine the margin of the neoplasm before ESD, revealing a clear unstained area within a large area of dark brown mucosa ( Fig. 3 ). Some columnar epithelial islands remained within the squamous metaplasia of the mucosa, with some unstained areas located outside the lesion. The lesion was completely resected without complications ( Fig. 4 ). Histopathology confirmed an intramucosal SCC infiltrating into the lamina propria (pT1a-M2) ( Fig. 5 ).

Endoscopic submucosal dissection (ESD) for early squamous cell carcinoma (SCC) extending from the cardia to the fundus.Video 1

Early SCC extending from the cardia to the fundus is an exceedingly rare type of gastric cancer. Smoking and the presence of gastric squamous metaplasia are often related to SCC. The cause of SCC may be related to long-term chronic inflammation of the gastric squamous-columnar junction, where normal columnar epithelium is replaced by squamous epithelium and then undergoes malignant change. This case confirms the relationship between SCC and squamous metaplasia of the gastric mucosa.

Endoscopy_UCTN_Code_CCL_1AB_2AD_3AB

